# Host modulation therapy in periodontitis, diagnosis and treatment—status update

**DOI:** 10.3389/fdmed.2024.1423401

**Published:** 2024-07-16

**Authors:** Lorne M. Golub, Hsi-Ming Lee, Joseph Bacigalupo, Ying Gu

**Affiliations:** ^1^ Department of Oral Biology and Pathology, School of Dental Medicine, Stony Brook University, Stony Brook, NY, United States; ^2^ Private Practice, Hempstead, NY, United States; ^3^ Department of General Dentistry, School of Dental Medicine, Stony Brook University, Stony Brook, NY, United States

**Keywords:** host modulation therapy, periodontitis, diagnosis, treatment, inflammation

The microbial biofilm (dental plaque) has long been known to initiate and perpetuate the inflammatory response in periodontitis. Anaerobic micro-organisms in the biofilm (especially *Porphyromonas gingivalis, Tannerella Forsythia,* and *Prevotella intermedia*), and their release of endotoxin and other bacterial products, have long been investigated as causative agents of periodontal inflammation and connective tissue breakdown including alveolar bone loss (1–4).

However, a recent strategy to manage periodontitis, as an adjunct to controlling the microbial biofilm (involving daily optimal oral hygiene, and scaling and root planing (SRP) at regular intervals), has been called “Host-Modulation Therapy (HMT)”. The concept of HMT was first introduced in the early 1980's (5); and later extensively discussed by Williams (6) and Golub in the 1990s (7). While periodontitis is initiated by specific anaerobic bacterial, the outcome of the disease is determined by the host response to the periodontal inflammation. As a result of these early discoveries, the treatment of periodontitis now includes two prongs: antimicrobial treatment (including SRP) and HMT which targets the host inflammatory responses, such as elevated levels of matrix metalloproteinases and inflammatory cytokines. One HMT approach involves the use of aspirin-like drugs or NSAIDs such as ibuprofen, flurbiprofen, etc (8).. However, the long-term use of these drugs poses risks for developing ulcers and other side-effects, and this approach is not discussed herein (8, 9).

More than 4-decades ago, Golub et al. (1983) (5) proposed, and then experimentally demonstrated, that tetracyclines, and by mechanisms unrelated to their use as broad-spectrum antibiotics, can effectively modulate the host response (10). The initial studies on experimental animals showed that minocycline can effectively reduce pathologically-excessive gingival collagenolytic activity and alveolar bone loss during diabetes in either conventional or germ-free rats (5), later studies showed more robust HMT efficacy with doxycycline (10).

Specifically, these investigators discovered that tetracyclines, unexpectedly, can inhibit the mammalian (host) cell production, and the extracellular activity, of host-generated matrix metalloproteinases (MMPs), specifically the collagenases, gelatinases, and macrophage metallo-elastase (10, 11). These proteolytic enzymes, produced by human and animal tissues, mediate the degradation and breakdown of the collagen fibers and other connective tissue constituents of the periodontium (proteoglycan ground substance, etc,) including the gingiva, the periodontal ligament, and the organic matrix of the alveolar bone (8).

This discovery ultimately led to the development of MMP-inhibitory host modulatory drugs for both dentistry (Periostat®) and medicine (Oracea®), both of which are government-approved (Food and Drug Administration; FDA) drugs requiring prescriptions for clinical use. Periostat® can be prescribed for treating periodontitis, as an adjunct to scaling and root planing and oral hygiene instruction. (Of interest, a short communication titled “Managing Tissue Health Around Natural Teeth And Dental Implants: A Clinician's Experience” described the use of Periostat® in approximately 800 patients, which appeared to reduce the severity of periodontitis and peri-implantitis (12). Oracea® is prescribed for the treatment of the common inflammatory skin diseases, acne and rosacea with significant improvement in both diseases (10, 11, 13).

The rationale behind the development of these drugs was not obvious (i.e., efficacy of a NON-antibiotic dose of the antibiotic, doxycycline), and these regimens were demonstrated to be safe and effective in clinical studies (8). As a result, these drugs were patented, government (FDA)-approved (after extensive doubleblind, placebo-controlled clinical trials), and made commercially available to dental and medical practitioners (dermatologists have been the major prescribers). Another strategy in the development of tetracyclines as “Host-Modulators” was to chemically modify the tetracycline molecule by eliminating the side-chain responsible for its antibiotic activity, i.e., the dimethyl amino group on carbon-4 (10, 11, 13). This modification resulted in the development of a series of chemically-modified tetracyclines (CMTs), one of which, CMT-3 (6-demethy 6-deoxy 4-dedimethy amino tetracycline; Incyclinide), has been tested in patients with a type of cancer, Kaposi's sarcoma (14). In the preliminary studies, these patients were found to respond favorably, exhibiting a 44% reduction in angiogenic lesions in their skin. However, in clinical trials a significant side-effect of this novel orally-administered compound emerged, namely an increase in sunburn (14), which resulted in a cessation of further testing of this CMT. Currently, additional research continues with the goal of developing newer CMTs which: (i) lack antibiotic activity in order to prevent the side-effect of the emergence of antibiotic-resistant micro-organisms; (ii) are potent MMP-inhibitors; and (iii) exhibit few, if any, side-effects such as increased sensitivity to sunburn.

Recently, newer HMT agents have been developed and studied as adjunctive treatments for periodontitis. Curcumin, a natural spice used widely in India cuisine, has been shown to exhibit anti-inflammatory properties. However, a significant deficit is its limited bioavailability. To overcome this problem, a novel group of chemically modified curcumins (CMCs) has been developed to increase its bioavailability and to enhance its anti-inflammatory activity (15). Research studies, involving *in vitro* enzyme assays, cell culture studies, and *in vivo* animal experiments, identified a lead compound, namely CMC 2.24, a phenylamino carbonyl curcumin. This novel compound, CMC 2.24, has demonstrated a significant effect on reducing pathologic elevated MMP levels, and reducing alveolar bone loss in animal models of periodontitis (16, 17). Moreover, CMC 2.24 can reduce the elevated MMP-9 levels (a biomarker for inflammatory periodontal and other diseases) in gingiva and plasma, before any visible clinical signs of periodontitis are observed (18). While investigating possible underlying mechanisms, in addition to its calcium and zinc binding *β*-diketone moiety that is similar to tetracyclines, CMC has shown a resolving-like activity to promote M2 macrophage polarization, therefore attenuating pathologic alveolar bone loss (19). These findings strongly suggest that CMCs can be developed as novel therapeutic HMT agents for treating periodontitis and other diseases, including arthritis, cardiovascular disease, and cancer.

As the concept of HMT has become widely accepted in the field, emerging HMTs have been studied as adjunctive therapies for periodontal diseases. In addition to CMTs and CMCs mentioned earlier, other potential host modulation therapies have been developed and studied by other groups. For example, anti-cytokine therapy utilizes monoclonal antibodies against inflammatory cytokines. Infliximab and Etanercept were developed as anti-TNF-α drugs and showed significant improvement in periodontal disease parameters (9). Studies have also demonstrated that probiotics can be a potential HMT in the management of periodontitis. Lactobacillus based probiotics revealed therapeutic effects in improving periodontal conditions (9, 20). Omega-3 polyunsaturated fatty acids and resolvins are another group of HMTs that have been studied extensively by Van Dyke et al. (21). These compounds do not inhibit acute inflammation, which is required for normal wound healing, but do prevent chronicity which would negatively affect wound healing (21). In animal and human clinical studies, Resolvins demonstrated great potential to reduce the severity of periodontitis (22, 23). As more innovations in HMT research emerge, a variety of potential HMT agents are being developed including stem cells (24), and complement inhibitors (25). Research has demonstrated that human periodontal stem cells can modulate inflammatory responses (24), and bone marrow-derived mesenchymal stem cells have shown great potential for periodontal regeneration in animal models (26). HMT targeting the complement C3 has been shown to reduce periodontal inflammation suggesting a therapeutic potential in treating peri-implantitis (25).

With emerging development of additional host modulation agents, as adjunctive therapy to existing periodontal approaches, plus the new diagnostic concepts developed by Sorsa et al, ie, the aMMP-8 chairside test for gingival crevicular fluid and periimplantitis sulcular fluid (27), HMT can greatly contribute to long-term improved periodontal outcomes.
